# Spatial transcriptomics in Alzheimer's disease: technologies, challenges and discoveries

**DOI:** 10.1186/s44477-026-00036-x

**Published:** 2026-06-16

**Authors:** Christina Huan Shi, Juan C. Piña-Crespo, Kevin Y. Yip, Timothy Y. Huang

**Affiliations:** 1 Center for Neurologic Diseases, Sanford Burnham Prebys Medical Discovery Institute, La Jolla, CA 92037 USA; 2 Center for Data Science and Artificial Intelligence, Sanford Burnham Prebys Medical Discovery Institute, La Jolla, CA 92037 USA; 3 Cancer Genome and Epigenetics Program, NCI-Designated Cancer Center, Sanford Burnham Prebys Medical Discovery Institute, La Jolla, CA 92037 USA

**Keywords:** Alzheimer’s disease, Spatial transcriptomics, Regional vulnerability and resilience, Cellular response to AD pathology, Pathological AD microenvironment, Cell–cell interaction, Aβ plaques, Tau

## Abstract

Alzheimer’s disease (AD) is a complex neurodegenerative disorder that is associated with cognitive decline in the elderly. While β-amyloid (Aβ) plaques and neurofibrillary tau tangles have been used to define and stage AD onset in human brain, how these pathologies can affect various cell types nearby remains a subject of intense interest in the field. Recent developments in spatial transcriptomic technology have seen accelerated growth, and spatial transcriptomic platforms have been used independently or together with single cell transcriptomic methods to characterize cellular changes in the AD brain from mouse models and human. Here, we review current-era spatial transcriptomic technologies, analytical pipelines and their implementation in AD research. We summarize findings from spatial transcriptomics in AD, and discuss limitations and challenges associated with various spatial transcriptomic platforms. The pathological hallmarks for AD were described by Alois Alzheimer over a century ago; from the convergence of a century of AD research and technological advances in imaging and transcriptomics, a new era in AD has emerged. Although current-era spatial platforms feature limitations and challenges, evolution of spatial transcriptomics and its combined implementation with other data modalities promises significant strides in AD and related neurodegenerative disorders.

## Introduction

Alzheimer’s disease (AD) is a progressive age-dependent dementia disorder that is associated with the accumulation of extracellular β-amyloid (Aβ) plaques and intracellular neurofibrillary tangles (NFTs) comprising hyperphosphorylated tau [1, 2]. The accumulation of Aβ and tau in the human brain follow distinctive spatial and temporal characteristics during aging and AD onset, where Aβ typically accumulates within the precuneus and cortical regions within the brain at least a decade or more prior to the manifestation of clinical symptoms [3]. Tau accumulation, however, correlates more closely with cognitive impairment during AD onset [4, 5]. Interestingly, tau pathology can appear in brain regions such as the entorhinal cortex and temporal lobe in normal aging in the absence of cognitive effects, and is a primary age-related tauopathy (PART) that is largely independent of memory impairment and Aβ pathology [6]. Cumulative pathological evidence indicates that cognitive decline correlates with the dispersion of tau pathology from the entorhinal cortex into neocortical regions [7, 8]. This suggests that the spatial distribution and temporal sequence of pathological progression during AD onset embody distinct features that could indicate stage, severity and cellular states in AD. In line with this, Braak staging which has been used to define the clinical progression of AD pathology is based on the dispersion of neurofibrillary tau tangles from the transentorhinal region to limbic regions and the hippocampus at mid-stages, and spread to neocortical regions at late stages [9]. Because significant advanced-stage AD pathology is observed in a proportion of cognitively normal individuals [10, 11], this has spawned a growing concept in the field based on the idea that some individuals may be resistant to Aβ and/or tau toxicity [12]. Although specific mechanisms underlying how individuals with normal cognition can overcome AD-associated proteotoxicity are still unclear, concepts such as “cognitive reserve” describe enhanced brain function and efficiency which may enable individuals to sustain cognitive function under pathological conditions [13]. Other concepts include “brain reserve” or “brain resilience” where enhanced anatomical features related to synaptic function or circuitry can confer resistance to degeneration [14]. Thus, in addition to pathology-related features that may define clinical decline in AD, unique characteristics may be seen in cognitively normal individuals with AD pathology potentially due to resilience mechanisms.

One central question in AD is how progressive accumulation of Aβ and tau pathology affects cellular behavior and function in AD brain. Early bulk RNA-seq studies have defined changes in transcriptomic profiles in AD and healthy brain from varying regions and progressive pathological states [15]. Single-cell RNA-seq (scRNA-seq; used here to collectively refer to both single-cell and single-nucleus RNA sequencing unless otherwise specified) studies have also characterized transcriptomic changes in various cell types in varying brain regions [16–22]. While bulk RNA-seq [23] and scRNA-seq methods [24, 25] have seen considerable technological and methodological development and optimization in AD/neurodegeneration research during the last few years [26], these methods rely on coupling pre-characterization of pathological features with transcriptomic analysis. With the development and use of spatial transcriptomic technologies to characterize changes in healthy and AD brain tissue, cellular expression profiles can be established in direct relation to pathological neurodegenerative landmarks in brain. Here we discuss the implementation, development and challenges related to spatial transcriptomic analysis in AD.

## An overview of spatial transcriptomics in the current era

**Fig. 1:**
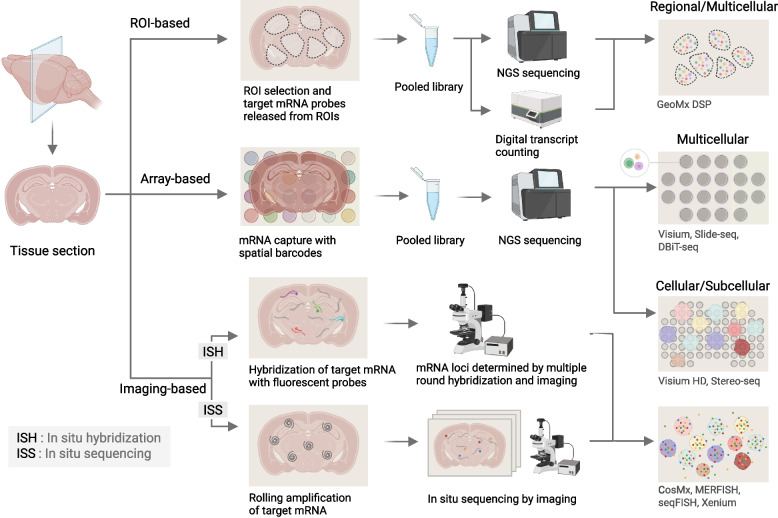
ROI-based, array-based and imaging-based spatial transcriptomic technologies. In ROI-based technologies, custom ROIs are selected and target mRNA probes are released from ROIs. The pooled library is profiled by NGS platforms or digital transcript counting platforms such as the nCounter platform from Bruker. ROI-based technologies generate regional or multicellular data. In array-based technologies, a tissue section is placed on a slide or spot/bead/nanoball array containing spatial barcodes, and captures mRNAs with labeled spatial barcodes. The pooled library is then sequenced by NGS platforms, and each transcript is mapped back to specific spatial coordinates on the tissue using spatial barcodes. Depending on the distance between adjacent spots and how the data is aggregated during analysis, array or barcode-based technologies usually yield multicellular data (e.g., Visium, Slide-seq, DBiT-seq), yet can also achieve cellular or subcellular resolution (e.g., Visium HD, Stereo-seq data; representation of cells detected in spots shown). Imaging-based technologies can detect mRNA *in situ* using either ISH or ISS. ISH approaches hybridize mRNA targets with fluorescent probes, and usually determine mRNA loci by multiple rounds of hybridization and imaging. ISS approaches directly read the transcript sequence *in situ* by imaging after rolling amplification of the target mRNA. Both ISH (e.g., CosMx, MERFISH, seqFISH, Xenium) and ISS (e.g., STARmap) generate data at subcellular resolution. Figures were created using BioRender

Spatial transcriptomics is used to profile mRNA expression in a spatial context, either at small-bulk or single-cell resolution. Use of hybridization methods to visualize DNA-RNA hybrids *in situ* with radioactive RNA dates back to the late 1960 s [27, 28]. Thanks to the commercialization of various spatial transcriptomics platforms, a surge in implementation of current-era spatial transcriptomics is seen ~ 2020 [29]. Based on how spatial information is preserved, current-era spatial transcriptomic methods fall into five categories including: (i) methods involving tissue microdissection or region of interest (ROI) selection, (ii) methods that preserve relative local positions followed by de novo construction of global spatial positioning, (iii) methods using spatial barcoded-arrays with next-generation sequencing (NGS), (iv) *in situ* hybridization (ISH) and (v) *in situ* sequencing (ISS) methods [29] (Table 1). Many reviews provide in-depth summaries describing the development of spatial transcriptomic technologies [29–35] as well as analysis [33, 35–38]. Here, we focus on the latter three categories of current-era spatial transcriptomic methods (Fig. 1, Table 1), which can profile a large number of spots or cells with either high gene throughput or high spatial resolution, and discuss their implementation in characterizing AD brain.

**Table 1:** Overview of representative and commercialized spatial transcriptomic platforms

Technology	Detection method^1^	Resolution	Multiplexity	Company	Gene panel	FFPE compatibility	Histological staining compatibility^2^
GeoMx DSP	ROI-based with NGS or nCounter^3^	Custom ROI sizes down to 10 μm	Up to ~ 20,000 genes	Bruker (previously NanoString)	Up to 400 custom RNA targets	Yes	H&E after ST (recommended); IF before ST;
Visium	Array/barcode with NGS	55 μm spots; 2 μm grid in Visium HD;	Whole transcriptome	10 × Genomics	N/A	Yes	Optional between H&E and IF; Before ST;
Slide-seq/Slide-seq V2	Array/barcode with NGS	Bead array (10 μm)	Whole transcriptome	Takara Bio (previously Curio)	N/A	No*	Not validated;
DBiT-seq	Array/barcode with NGS	Microfluidic barcoding (10–50 μm/pixel)	Whole transcriptome	AtlasXomics	N/A	Yes	IF; Before ST;
Stereo-seq/Stereo-seq V2	Array/barcode with NGS	Nanoball array (~ 0.5 μm)	Whole transcriptome	BGI/STOmics	N/A	Yes	H&E and IF; Before ST;
CosMx	ISH	Subcellular (0.12 μm/pixel)	1,000 to 6,000 genes	Bruker (previously NanoString)	Up to 50 custom genes for the 1 K gene panel; Up to 200 custom genes for the 6 K gene panel;	Yes	H&E and IF; After ST;
MERFISH	ISH	Subcellular (0.1 μm/pixel)	140, 300, 500 or 1,000 genes	Vizgen	Full custom panel	Yes	H&E—After ST; IF—Before or after ST;
seqFISH/seqFISH +	ISH	Subcellular (~ 0.2 μm/pixel)	Up to ~ 10,000 genes	Spatial Genomics	Allows custom gene panels	Not validated	Not validated
Xenium	ISH	Subcellular (0.2 μm/pixel)	Up to 5,000 genes	10 × Genomics	Up to 100 custom genes to existing panels; Up to 480 genes for a fully custom panel;	Yes	H&E and IF; After ST;

^*^No FFPE compatibility with Curio Slide-seq platforms

^1^NGS-next generation sequencing, ISH-*in-situ* hybridization, ISS-*in situ* sequencing

^2^H&E-Hematoxylin and Eosin, IF-immunofluorescence, ST-spatial transcriptomics

^3^nCounter is a molecular profiling platform originally developed by NanoString (acquired by Bruker Spatial Biology in 2024) that performs direct digital counting of nucleic acids or proteins using fluorescent barcodes, without sequencing or PCR amplification. nCounter can simultaneously profile around 800 mRNA targets

### Current-era array-based and imaging-based spatial transcriptomic technologies

Spatial barcoded-arrays with NGS, or array-based spatial transcriptomic approaches, capture mRNA in spatially-divided spots, beads or bins using spatially barcoded arrays, and are well-suited for transcriptome-wide profiling (Fig. 1, Table 1). Each spot typically captures multiple cells, and therefore does not produce profiles at single-cell resolution. Currently, 10 × Genomics Visium, developed based on an early array-based spatial transcriptomics method [39], is the most widely used commercial array-based platform which offers whole-transcriptomic analysis of spots of 55 μm in diameter, with each spot typically containing 1–10 cells. In 2024, 10 × Genomics released Visium HD [40] which offers increased resolution (2 μm × 2 μm barcoded squares). In addition to Visium, other representative barcode-based or array-based spatial transcriptomic approaches include Slide-seq and the upgraded version, Slide-seqV2 [41, 42], “deterministic barcoding in tissue for spatial omics sequencing” (DBiT-seq) [43] and “SpaTial EnhancedResolution Omics-sequencing” (Stereo-seq) [44]. Slide-seq uses bead barcodes to capture whole-transcriptome with a spatial resolution of around 10 μm, and its detection efficiency was further improved in Slide-seqV2 with sensitivity approaching droplet-based single-cell RNA-seq methods. Instead of using a pre-patterned array, DBiT-seq generates spatial barcodes directly on tissue via microfluidic channels, and can profile the whole-transcriptome with a spatial resolution from 50 μm up to 10 μm per pixel. In Stereo-seq applications, the slide is patterned with DNA nanoballs (DNBs), each carrying a unique spatial barcode. Individual DNBs are around 220 nm in diameter, and spot center-to-center distance is around 500 to 715 nm, offering a cellular/subcellular resolution of around 500 nm. Due to the low gene detection rate in each DNB, Stereo-seq data is typically grouped into bins (e.g. Bin 50 aggregates every 50 × 50 DNBs yielding a bin size of 25 μm × 25 μm). In general, current array-based spatial transcriptomic methods can capture large tissue areas and enable whole-transcriptome profiling but typically measure transcripts from multiple cells per spatial spot. Although several high-resolution array-based platforms (e.g., Visium HD and Stereo-seq) have improved spatial resolution toward single-cell or subcellular levels, these platforms are also limited with respect to detection sensitivity (Table 2).

**Table 2:** Considerations with representative commercialized spatial transcriptomic platforms

	Array/barcode-based methods		Imaging-based methods
Representative platforms	Visium, Slide-seq, DBiT-seq	Visium HD, Stereo-seq	CosMx, MERFISH, seqFISH, Xenium
Spatial resolution	Multi-cellular	Single-cell or subcellular	Subcellular
Multiplexity	Whole transcriptome		Targeted genes (typically hundreds to thousands of genes)
Detection sensitivity	Low sensitivity, particularly for low-abundance genes		High sensitivity for targeted genes
Tissue throughput	High throughput with next generation sequencing (NGS)		Low throughput due to multiple imaging cycles
Experimental time and complexity	Comparatively rapid and standardized workflow		Longer and more complex processing protocols
Computational complexity and results interpretability	Lower preprocessing burden with sequences; Higher burden for deconvolution and integration with scRNA-seq data; Multi-cellular data complicates interpretation; Please see description (*) for single-cell or subcellular resolution array-based methods		Higher preprocessing burden with images; Lower burden for integration with scRNA-seq data; Cellular-level analysis enables cell type-specific interpretation
Use cases	Discovery-based studies		Targeted, validation-based studies

^*^For high-resolution array- or barcode-based platforms, such as Visium HD and Stereo-seq, transcript capture remains sequencing-based and generally requires less intensive raw-data preprocessing than imaging-based methods. However, downstream analysis often involves a trade-off between spatial resolution and analytical complexity. Investigators may aggregate transcripts into larger spatial bins to improve effective sensitivity by increasing transcript counts per analytical unit and reducing sparsity, which can reintroduce multicellular signals and necessitate cell type deconvolution and complicate integration with scRNA-seq data. Alternatively, auxiliary imaging data can be used for cell segmentation to generate approximate single-cell profiles, although this introduces additional image-processing challenges

ISH and ISS are imaging-based spatial transcriptomic approaches that detect transcripts *in situ* and can achieve subcellular resolution (Fig. 1, Table 1). ISH methods detect target sequences by hybridization of complementary fluorescent probes, and ISS directly reads transcript sequences *in situ* (Fig. 1). Both ISH and ISS approaches use high-resolution imaging to spatially locate transcripts, together with additional staining to identify cell or nucleus boundaries. Single-cell gene expression profiles are reconstructed typically by assigning transcripts to cells based on their boundaries. Popular commercial imaging-based spatial transcriptomic platforms include CosMx from NanoString, Multiplexed error-robust FISH (MERFISH) [45] on the MERSCOPE platform from Vizgen, and Xenium from 10 × Genomics. CosMx, MERFISH and Xenium are ISH-based methods. CosMx can achieve subcellular resolution with a pixel-edge as small as 120 nm, and offers pre-designed gene panels of 1,000–6,000 genes, or even whole transcriptome panels as demonstrated in prototype. MERFISH on the MERSCOPE platform can achieve a pixel-edge length of 100 nm and offers gene panels of up to 1000 genes. Xenium also achieves subcellular resolution with a pixel-edge ~ 200 nm wide, and supports gene panels of up to 5,000 genes. Other representative imaging-based spatial transcriptomic approaches include ISH-based seqFISH [46] and seqFISH + [47], and ISS-based STARmap [48] platforms. In addition to custom probe design, some platforms provide pre-designed gene panels for specific applications, such as mouse neuroscience gene panel offered by CosMx and MERSCOPE. Although imaging-based spatial transcriptomic approaches can characterize cell-level gene expression profiles through the detection of transcripts at subcellular resolution, analysis using these methods is often limited to panels comprising several hundreds to thousands of genes. Capacity to capture profiles approaching the whole-transcriptome has been demonstrated for certain imaging-based spatial transcriptomic approaches [47, 49], however, their popularity is limited due to both technical and practical concerns including increased error rates due to optical crowding, increased probe-design complexity and costs. Additional challenges include prolonged experimental run time due to limited imaging throughput, and increased computational complexity of image preprocessing (Table 2) [50, 51]. Further limitations and considerations with array-based and imaging-based spatial transcriptomic technologies are discussed below (“Limitations, challenges and future directions with spatial transcriptomics”).

### Use and considerations for spatial transcriptomics in AD research

**Fig. 2:**
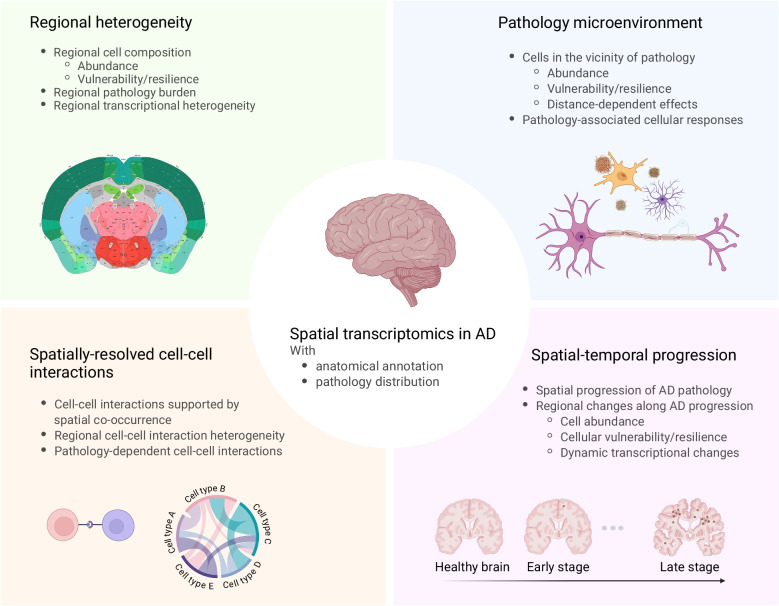
Spatial transcriptomics: practical applications in AD brain. Schematic indicating key applications associated with spatial transcriptomics in AD brain, including characterization of regional heterogeneity, pathology microenvironment, spatially-resolved cell–cell interactions, and spatial–temporal progression of AD pathology. Figures were created using BioRender

Spatial transcriptomics has experienced a recent boost in prevalence in AD research in both human AD brain and mouse AD models (Table 3), and can be used in a number of analytical applications (Fig. 2). Emergence of AD-related research using current-era spatial transcriptomic technologies from ~ 2020 continues to grow, thanks to the accessibility of commercialized spatial transcriptomic platforms. Although the total number of studies in AD using spatial transcriptomics is still limited, the field is seeing a shift from multicellular to subcellular-resolution spatial transcriptomic methods. This reflects expanding accessibility to various spatial transcriptomic platforms combined with the necessity to investigate AD pathobiology at the single-cell level.

**Table 3:** Examples of spatial transcriptomics in profiling human AD and mouse AD models

Study	Species or model/Brain region	Spatial transcriptomics platform
(Chen, Lu et al. 2020)	APP^NL−G−F^ (mouse)/whole coronal sections, human AD/superior frontal gyrus	spatial transcriptomics*, ISS
(Navarro, Croteau et al. 2020)	3xAD (3xTg AD) and 3xPB (3xAD/Polβ+/-) (mouse)/hippocampus, olfactory bulb	10 × Visium
(Chen, Chang et al. 2022)	Human AD/middle temporal gyrus	10 × Visium
(Castranio, Hasel et al. 2023)	PSAPP (mouse)/coronal hemispheres	10 × Visium
(Zeng, Huang et al. 2023)	TauPS2APP (mouse)/cortex, hippocampus	STARmap PLUS
(Zhang, Xia et al. 2023)	5xFAD (mouse)/coronal sections	Stereo-seq
(Lee, Devanney et al. 2023)	APOE3, APOE4 × 5xFAD (mouse)/coronal sections	10 × Visium
(Liu, Li et al. 2023)	APP/PS1 (mouse)/coronal hemispheres	10 × Visium
(Lu, Saibro-Girardi et al. 2023)	APP/PS1 (mouse)/coronal hemispheres	10 × Visium
(Choi, Lee et al. 2023)	5xFAD (mouse)/coronal hemispheres	10 × Visium
(Lee, Suh et al. 2024)	5xFAD (mouse)/coronal hemispheres	10 × Visium
(Gabitto, Travaglini et al. 2024)	Human AD (middle temporal gyrus)	MERFISH (140 gene panel)
(Miyoshi, Morabito et al. 2024)	Human AD, DS/frontal cortex; 5xFAD (mouse)/coronal hemispheres	10 × Visium
(Mallach, Zielonka et al. 2024)	APP^NL−G−F^ (mouse)/coronal hemispheres, hippocampus, cortical regions	CosMx, Stereo-seq
(Wang, Han et al. 2025)	Human AD/hippocampus	Stereo-seq
(Avey, Ng et al. 2025)	Human AD/DLPFC	10 × Visium
(Johnston, Berackey et al. 2025)	5xFAD, Trem2 R47H-5xFAD (mouse)/coronal sections	MERFISH (300 gene panel)
(Shen, Shen et al. 2025)	5xFAD (mouse)/coronal hemispheres	10 × Visium
(Gong, Haeri et al. 2025)	Human AD/prefrontal cortex	Stereo-seq
(Olst, Simonton et al. 2025)	Human AD/frontal cortex, temporal cortex, parietal Cortex, hippocampus	10 × Visium/Visium HD
(Ji, Giles et al. 2025)	R955-hTau +/+ (mouse)/coronal hemispheres	10 × Visium
(Gaur, Bryois et al. 2025)	Human AD/temporal cortex	CARTANA ISS^1^ (155 gene panel)
(Karasik, Eger et al. 2025)	5xFAD (mouse)/hippocampus	ExSeq (ISS) [PMCID: PMC7900882] (101 gene panel)
(Bayaraa, Aksu et al. 2026)	Human AD/entorhinal cortex, occipitotemporal cortex, dorsolateral prefrontal cortex, occipitotemporal cortex	10 × Visium
(Dharshini, Sanz-Ros et al. 2026)	Human AD/prefrontal cortex, precuneus, primary visual cortex, motor cortex, entorhinal cortex, and hippocampus	10 × Visium/Xenium
(Gelber, Romero et al. 2026)	APP23 (mouse)/sagittal sections	10 × Visium

^*^spatial transcriptomics refers to methods used in (Ståhl, Salmen et al. 2016), which was acquired by 10 × Genomics in 2018 and commercialized as the Visium platform

^1^CARTANA in situ sequencing (ISS) was acquired by 10 × Genomics in 2020

Although substantial efforts are driving spatial transcriptomic technologies toward both subcellular resolution and whole-transcriptomic profiling, current well-established spatial transcriptomic platforms still see a tradeoff between resolution and multiplexity. In general, spatial transcriptomic platforms achieving subcellular resolution feature limited multiplexity of up to several thousand genes, which limits the depth of downstream analysis. Conversely, whole-transcriptomic spatial transcriptomic platforms generally lack subcellular resolution, and do not support analysis at single-cell resolution (Table 2). To address these limitations, scRNA-seq is often used concurrently to impute whole-transcriptomic profiles of spatial transcriptomic datasets at cellular resolution; alternately, spatial organization of cells profiled by scRNA-seq can be computationally inferred by mapping to spatial transcriptomic profiles based on similarities in gene expression. In this context, scRNA-seq and spatial transcriptomic datasets generated from adjacent tissue sections are advantageous as they often share similar cell populations, tissue architecture, pathology deposition and cellular states. Thus, scRNA-seq and spatial transcriptomic data were frequently combined to additively enable in-depth whole transcriptome analysis at single cell resolution with spatial context [52–57].

Given that brain tissue preservation typically involves formalin fixation and paraffin embedding (FFPE), compatibility of spatial transcriptomic methods with FFPE tissues is critically important (Table 1). Unlike mouse brain tissues which are typically fresh-frozen with relatively high RNA quality, human brain samples are commonly processed as FFPE blocks and require specialized library preparation procedures that accommodate RNA crosslinking and fragmentation resulting from formalin fixation, paraffin embedding and long-term storage. Therefore, compatibility of spatial transcriptomic procedures with FFPE samples is essential. The Visium platform employs a probe-based chemistry pipeline that uses specifically-designed probes to capture degraded RNA fragments; however, detection is limited to protein-coding genes. Similarly, MERFISH incorporates additional steps for FFPE samples including deparaffinization, decrosslinking, and other steps to preserve fragmented RNAs and improve probe hybridization to RNA fragments.

Compatibility of histological staining on the same tissue section is another important consideration for spatial transcriptomic experiments, particularly in AD studies as it enables direct analysis of transcriptional changes within pathological microenvironment (Table 1). Immunofluorescence (IF) is widely used for pathology staining due to its high specificity and resolution. And it is compatible with most current-era spatial transcriptomic platforms. For ROI-based platforms such as the GeoMx Digital Spatial Profiler (DSP), IF staining is integrated into its workflow and performed before transcript profiling to guide ROI selection. For array-based platforms including Visium, DBiT-seq and Stereo-seq, IF staining is performed before transcript acquisition. For imaging-based platforms including CosMx and Xenium, IF staining is typically performed after transcriptomic profiling, whereas MERFISH allows IF staining either before or after transcriptomic profiling (Table 1).

In addition to resolution, multiplexity, FFPE compatibility and pathology-staining compatibility, spatial transcriptomic platform selection also involves other considerations including tissue coverage, scalability, throughput, and cost, as well as software to support raw data processing and preliminary data quality assessment [58, 59].

## Processing and analyzing spatial transcriptomics datasets in AD

Spatial transcriptomics and scRNA-seq share many analytical computation modules, and routine pipelines for both data types have been extensively reviewed [33, 35–38, 60–63]. This section briefly describes standard spatial transcriptomic data processing procedures with a particular focus on adaptations required for brain tissues in AD research.

### Cell segmentation in spatial transcriptomic datasets

Analysis of gene expression profiles at the cellular level in spatial transcriptomic datasets requires preparatory steps to define and annotate cell boundaries through cell segmentation. In this review, cell boundary is used as a generic term referring to either cell boundary or nuclear boundary. Based on cell boundaries, spatially-defined transcripts are then assigned to cells, and aggregated to generate gene expression profiles for each cell. Cell segmentation is typically based on morphological images [64–68], transcript distribution [69, 70], or both [70–73].

Cell segmentation in brain is particularly challenging due to high cell density, heterogeneous cell morphology, and substantial background signals from neuropil and AD pathologies. The brain comprises densely-packed cells, resulting in overlapping cell bodies which can hinder accurate cell segmentation. Computational methods can be used to identify inaccuracies due to overlapping cells based on gene expression profiles [74] or imaging features [75]. Given the substantial morphological diversity of brain cell types, use of a single model to perform cell segmentation across all cell types may be challenging; use of specialized segmentation models that account for morphological differences across brain cell types may improve segmentation accuracy. Additionally, excessive background signals from AD pathology, as well as neuropils comprising a dense network of axons, dendrites, synapses and glial processes interspersed between cell bodies can also complicate cell segmentation. These challenges may be further exacerbated in regions with high pathological burden, particularly in late-stage AD brain tissue; for example, DAPI which primarily stains nuclei, was found to also stain Aβ plaques [76]. To address issues related to background noise, staining strategies can be optimized to reduce false positive signals and improve segmentation accuracy [77, 78]. For example, a combinatorial staining strategy (e.g., DAPI + polyT) can improve cell segmentation accuracy compared to single staining alone [77]. In addition, existing machine learning models can be further refined using a “human-in-the-loop” approach where user-annotated or adjusted cell boundaries can be incorporated to improve segmentation performance [67, 68].

### Data processing in spatial transcriptomics

As spatial transcriptomic and scRNA-seq data both comprise cell/spot × gene matrices, they share many core data processing steps, including general data quality control, normalization, clustering and cell type annotation. Toolkits or libraries for spatial transcriptomic data analysis include Seurat [79] and Scanpy [80], which were originally designed primarily for scRNA-seq analysis and later extended to support spatial transcriptomic datasets. Other analysis toolkits specifically for spatial transcriptomic data analysis are also available [81–83]. This section discusses data processing procedures unique to spatial transcriptomics and used in studies related to AD.

Similar to doublet artifacts in scRNA-seq datasets where two cells are sequenced as a single cell, inaccurate segmentation of neighboring or overlapping cells can compromise downstream spatial transcriptomics analyses. These artifacts can be inferred using gene expression-based or image-based approaches. In addition, data pertaining to cell size acquired during cell segmentation in spatial transcriptomics can be used to remove abnormally small or irregularly large and potentially overlapping cells due to inaccurate segmentation. Filtering such artifacts improves the accuracy of downstream analyses.

To normalize transcript levels in spatial transcriptomic data, common strategies include global scaling using the total number of transcripts per cell, use of cell-specific scaling factors estimated by pooling similar cells together to handle heterogeneous dropouts [84], applying variance-stabilizing transformations [85], and using cell area or volume as scaling factors [86]. Using cell area or volume was found to be more suitable for spatial transcriptomic datasets with skewed or limited gene panels since overall transcript abundance cannot be accurately estimated from a small number of genes [87].

Clustering and annotation of spatial transcriptomic datasets can employ different approaches depending on both data resolution and specific endpoints required. Clustering based on gene expression profiles is often used for identifying cell types and subtypes by examining top differentially expressed genes (DEG) within each cluster or through marker gene expression. Joint clustering of gene expression profiles and spatial coordinates can be used to identify spatial tissue domains or anatomical structures [88–91]. Annotation of cells at single-cell resolution in spatial transcriptomics can be inferred by knowledge-guided approaches using cell marker genes, or by mapping annotated reference data to spatial transcriptomics data to infer cell types which typically use all available genes instead of only depending on marker genes [92, 93]. For multicellular spatial transcriptomic datasets, cell type composition in spots/bins/beads can be estimated using reference-based approaches [94, 95], as well as reference-free methods [96]. scRNA-seq datasets from large atlas databases, such as mouse brain datasets from Allen brain cell atlas [86] and human brain cell datasets from the BRAIN Initiative Cell Census Network (BICCN) [97], provide valuable references to annotate brain cell types in spatial transcriptomics.

### Anatomical annotation of brain regions or subregions

Spatial transcriptomic profiles in brain can be annotated into anatomical regions or subregions for region-specific analysis and comparisons across different regions [52, 76, 98–109] (Table 3). To this end, brain slices are often aligned to a common coordinate framework (CCF) of an anatomy-defined high-resolution 3D brain map, such as the Allen Mouse Brain CCF [110]. CCF alignment typically uses histological images such as Hematoxylin and Eosin (H&E)-stained images, anatomical landmarks, and cytoarchitectural features such as cell morphology and density. In addition, anatomical region-specific molecular features (e.g., gene expression and protein markers) can be used to supplement CCF mapping or guide annotation of an anatomical region. For example, cortical layers can be annotated using layer-specific marker genes, together with H&E and IF staining without using a defined CCF [100]. Both CCF-based and region-specific marker gene-based approaches depend heavily on prior knowledge pertaining to anatomical brain characteristics and region-specific features. De novo approaches have also been used to segment anatomical regions using only transcriptomic expression profiles and their spatial coordinates. For example, joint clustering of transcriptomic profiles and spatial coordinates using BayesSpace [88] can effectively annotate cortical layers and white matter (WM) with high consistency [52]. Anatomical annotation of brain regions in spatial transcriptomics enables characterization of region-specific molecular, cellular, and pathological heterogeneity in AD brain.

### CCI inference with spatial context

Various databases comprising curated ligand/receptor binding pairs such as CellPhoneDB [111], CellChatDB [112], LRIDB [113] and CITEdb [114] have been established. Cell–cell interaction (CCI) analysis is widely performed on scRNA-seq data, and potential CCIs are inferred solely based on enrichment of ligand or receptor gene expression [112, 115–117]. The spatial context provided in spatial transcriptomic data can be used as additional information to infer CCIs [115, 116, 118–122] supported by spatial co-localization or potential direct physical contact which is essential for classical ligand-receptor signaling.

### Pathology staining, segmentation and quantification

Recent advances in spatial transcriptomics have enabled discoveries directly linking spatial transcriptomic profiles with pathological features such as Aβ plaques and tau pathology [52, 76, 98, 100–102, 104–106, 123–127]. AD pathology is usually stained and imaged concurrently on tissues used for spatial transcriptomic analysis, or stained and imaged on adjacent brain slices. Many spatial transcriptomic platforms such as Visium, DBiT-seq, Stereo-seq, CosMx, MERFISH and Xenium support pathology staining on the same tissue sections used for spatial transcriptomic data acquisition.

While the majority of published AD studies using spatial transcriptomics so far have characterized gene expression profiles in proximity to Aβ [52, 76, 98, 102, 104–106, 123, 125]; relatively fewer studies consider Aβ and tau pathology in combination [100, 101]. Various antibodies have been used to characterize Aβ plaque deposition in spatial transcriptomic applications such as 6E10 [98, 104, 105, 123], MOAB-2 [124], 4G8 [125, 128], or Aβ 6F/3D (M08762) monoclonal antibodies [106]. Aβ plaques have also been stained using dye-based stains such as the X-34 Congo red derivative [101, 102] or Amylo-Glo [52] in spatial transcriptomic studies. So far, most spatial transcriptomic studies characterizing tau pathology use the AT8 phosphorylated tau (ptau) antibody as an indicator of pathological tau phosphorylation [100, 101]. The AT8 antibody detects pS202/T205 [129], and pS208 [130] tau phosphoepitopes which are commonly seen in tau paired-helical filaments. Strong AT8 ptau signals are observed in AD brain, with relatively less reactivity in healthy adult brain by western blotting [131–133]. Phosphoproteomic analysis indicates that phosphopeptides containing the AT8 pS202/T205 phosphoepitope can be detected in a majority of AD cases, as well as in some (~ 20–40%) control cases, potentially indicating that AT8 ptau may be phosphorylated in prodromal phases of onset [134]. This suggests that variability in AT8 ptau detection may arise between individuals and cohorts, as well as detection methods.

While identification and quantification of Aβ and tau pathology in stained images previously involved manual annotation methods [100, 104], computational methods are now widely used. Custom pipelines using image analysis software are widely implemented in spatial transcriptomics [52, 98, 101, 105, 125]. For example, histogram-based threshold methods have been implemented in ImageJ to generate binary annotations for Aβ plaques [98]. Automated custom imaging analysis protocols to segment Aβ have also been applied using the NIS-Elements software package from Nikon [52], and computational pipelines have been used to quantify ptau intensity using Fiji [101]. In addition to the integrated use of image analysis software, computational methods utilizing image analysis algorithms [106] and machine learning methods [124] can also be implemented.

Aβ and tau pathology significantly differ with respect to spatial distribution, biological characteristics and morphological features. Thus, differing methods are required for identifying, annotating and quantifying Aβ and tau pathology. Given that Aβ plaques form compact extracellular structures, most methods segment individual Aβ plaques [52, 76, 100, 101, 104–106, 124, 125]. Alternatively, some studies annotate Aβ plaques using an overall intensity score within a small region, such as cell body area [98, 102]. In contrast to Aβ, tau pathology is largely intracellular and manifests in neurons as small and irregular structures. Therefore, methods to annotate and quantify tau pathology is primarily based on staining signal intensity [100, 101, 106]. Spatial annotation and quantification of AD pathology can then be integrated with anatomically mapped spatial transcriptomic profiles to characterize pathology-related regional features, including regional pathology burden, transcriptomic heterogeneity across regions with pathology, local pathological microenvironment, and associated cell–cell crosstalk (Fig. 2).

### Combining and integrating spatial transcriptomic and scRNA-seq datasets

Whole-transcriptome, matched scRNA-seq profiles are often used in parallel with spatial transcriptomics [52, 53, 104, 106, 125], thereby complementing and providing cross-validation between platforms in brain studies related to AD. scRNA-seq can serve differing roles in spatial transcriptomics depending on the resolution and multiplexity of the spatial transcriptomics dataset.

For high-resolution single-cell spatial transcriptomics datasets probed with a limited gene panel, scRNA-seq data can be used as a reference to design a probe panel which can effectively distinguish different cell populations [86, 135]. In addition to guided selection of cell markers, scRNA-seq can be used to aid and optimize gene selection in probing specific biological pathways or processes [76]. In these instances, spatial transcriptomics is primarily used to investigate or validate specific hypotheses derived from scRNA-seq data within a spatially resolved context. scRNA-seq data is also widely used as a reference to annotate cell types in spatial transcriptomics [106]. In addition, scRNA-seq profiles can be mapped to spatial transcriptomics datasets to impute whole-transcriptomic gene expression for each cell, or alternatively, spatial organization of cells profiled by scRNA-seq can be inferred using spatial transcriptomic data [86, 135].

For low-resolution multi-cellular spatial transcriptomic datasets, scRNA-seq profiles can be used to infer cell type composition for “cell type-aware” analyses involving cell abundance and gene expression changes [94, 95]. Multicellular spatial transcriptomics can also be used to infer spatial organization of cells in scRNA-seq datasets [52]. For example, snRNA-seq profiles can be mapped to multicellular spatial transcriptomic datasets by predicting spatial coordinates of each snRNA-seq cell with CellTrek [136], thereby consolidating whole-transcriptomic spatial profiles at single-cell resolution [52].

## Biological insights from spatial transcriptomics in AD brain

Spatial transcriptomics studies have enabled the characterization of healthy and diseased cellular states within intact tissue, and have provided insight into cellular gene expression profiles in relation to brain region, pathology, and neighboring cells. Here, we discuss recent work in AD using spatial transcriptomics (Table 3) focusing on current-era high-resolution or high-throughput platforms, and highlight their biological insights in AD brain. We apologize for the excellent studies in AD or AD-related dementia utilizing spatial transcriptomic methods outside the scope of this review.

### Regional vulnerability and resilience

A defining feature of AD is the selective vulnerability of specific brain regions and cell populations to aging-related stressors. Neurofibrillary tau pathology follows a highly stereotyped progression pattern defined by Braak staging, initiating in transentorhinal and entorhinal regions before spreading into the hippocampus and neocortex [9], whereas Aβ deposition originates in the precuneus and other cortical regions [3, 137]. Notably, substantial AD pathology can also be observed in cognitively normal individuals, raising important questions about mechanisms of resilience and resistance to neurodegeneration. However, molecular features that render specific brain regions and cell populations vulnerable or resistant to degeneration remain unclear. Given that spatial transcriptomics preserves anatomical organization while resolving regional and cell type-specific transcriptional states in relation to pathology, use of spatial transcriptomics is particularly well suited to addressing these questions.

Spatial transcriptomic studies have revealed substantial regional heterogeneity in both pathological burden and molecular responses across AD brain regions. In human hippocampus, increased deposition of Aβ plaques was observed in the CA1 region, coinciding with a reduction in CA1 neurons, as well as enrichment of microglia and astrocytes in proximity to Aβ plaques [106, 137]. Further Stereo-seq analysis coupled with snRNA-seq analysis revealed increased expression of genes related to synaptic pruning in the fimbria of AD, as well as increase in genes related to energy generation in the CA region [106]. Similarly in mouse hippocampus, *LPL* (lipoprotein lipase), a microglia disease-associated marker [138] that facilitates lipid and Aβ uptake and degradation [139], featured enhanced up-regulation in the CA1-3 subregions in 3xAD (3xTg AD, APP K670N/M671L, MAPT P301L, PS1 M146V) or 3xPB (3xTg AD/Polβ+/-) AD mouse models [99]. In frontal cortex and posterior cingulate cortex of the human brain, spatial transcriptomic analysis revealed downregulation of genes modules associated with neurotransmission, neurodevelopment and amyloid-β formation exclusively in white matter and cortical L3/L4 layers in early-stage AD [52].

Region- and cell type-specific depletion has also been described in AD brain, implicating selective vulnerability in neurons with disease progression and pathological burden. In human brain, neuronal subpopulations in upper cortical layers within the middle temporal gyrus (MTG) and Brodmann area 9 (A9) were found to be depleted with progressive onset of AD pathology [53]. A vulnerable neuronal Sst^+^ subpopulation, localized primarily to supergranular layer II and III, featured downregulation of specific kinases including tyrosine kinase and calcium^2+^/calmodulin-dependent kinase families, and E3 ubiquitin ligases in early stages of pathological onset compared to unaffected Sst^+^ subpopulations [53]. In human temporal cortex, Parvalbumin (*PVALB*) neurons were depleted in gray matter in late Braak stage [54]. Other studies have noted subregion-specific vulnerable cell types in human hippocampus [106]. In particular, excitatory neurons were depleted in CA1, CA2 and DG, but not in CA3/4 subregions in AD hippocampus, suggesting resistance of CA3/4 excitatory neurons to degeneration during AD onset [106].

In contrast, selective resilience and molecular signatures associated with resilience features have also been characterized using spatial transcriptomics. In human brain, a resilient excitatory neuron population co-expressing *RORB*, *CUX2*, and *EYA4* was identified in layer 4 of primary visual cortex (BA17) [57]. Although Layer 4 is associated with amyloid plaque deposition, it is also associated with reduced tau pathology, and is considered to comprise neurons resistant to degeneration [57]. Layer 4 neurons feature upregulated expression of *KCNIP4*, a voltage gated potassium channel–interacting protein associated with reduced excitotoxic hyperexcitability. Functional validation in an AD mouse model demonstrated that *KCNIP4* upregulation suppresses activity-dependent genes (Arc, c-Fos), consistent with decreased neuronal excitability [57]. This suggests that high *KCNIP4* expression attenuates neuronal firing and activity-dependent signaling, and potentially acts as a functional barrier limiting both intrinsic vulnerability and activity-dependent trans-synaptic propagation of tau pathology across connected circuits.

Together, these studies suggest that selective vulnerability and resilience in AD likely reflects interactions between intrinsic transcriptional programs, regional microenvironment, pathological burden, and circuit-level connectivity. Spatial transcriptomics has enabled AD research to progress beyond descriptive anatomical staging by identifying molecular programs associated with both vulnerability and resilience across various cell types and anatomically distinct brain regions.

### Spatially-defined cellular responses to AD pathology

While regional and cell type-specific vulnerability and resilience provides an important framework for understanding selective degeneration in AD, gene expression changes in direct proximity to Aβ and tau pathology may represent more immediate cellular responses to pathological stress. Spatial transcriptomics has enabled direct characterization of transcriptional changes within pathological microenvironments, revealing localized cellular responses that are difficult to resolve using dissociative approaches.

Early studies used spatial transcriptomics [39] to characterize cell-specific changes associated with Aβ in humanized APP^NL−G−F^ mouse models and identified 57 plaque-induced genes (PIGs) associated with the complement system, oxidative stress, lysosomes and inflammation [98]. Interestingly, further analysis of PIGs using ISS data [140] revealed that PIGs were largely derived from microglia, and to a smaller extent, astrocytes and other cell types [98]. These studies provided early evidence that Aβ plaques are associated with highly localized multicellular transcriptional responses.

Subsequent studies [101, 105, 123, 125] have made significant inroads characterizing changes in various cell populations in relation to Aβ pathology across brain regions. The role of glia in AD pathogenesis, microglia and astrocytes in particular, has become a subject of interest in AD pathogenesis [141, 142]. Microglia have an active role in sensing and clearing Aβ [143], and form a barrier around Aβ plaques, thereby limiting plaque diffusion [144]. Distinctive expression profiles comprising disease associated microglia (DAM) [138], or neurodegenerative microglia (MGnD) [145] signatures have been characterized in AD mouse models by snRNA-seq or bulk RNA-seq analysis in sorted microglia, which contrast from expression profiles in homeostatic microglia. Perhaps not surprisingly, spatial profiling methods have noted enrichment of microglia nearby plaques across brain regions in both human and mouse brains [76, 101, 105, 106]. For example, microglia were enriched around plaques in CA1 of human hippocampus [106]. In TauPS2APP mouse brain cortex and hippocampus, spatial profiling using STARmap PLUS identified enrichment of DAM microglia in proximity to plaques [101]. And the homeostatic gene *P2ry12* was found to be downregulated in Aβ-proximal microglia, reinforcing the notion that microglia are responsive to spatial distance to amyloid pathology [76]. Similar to microglia, astrocytes take up and clear Aβ [146, 147], and are found in proximity to Aβ plaques in human [148] and AD mouse brain [149]. As disease-associated astrocytes (DAA) signatures have also been characterized in AD and other pathological mouse models [150], spatial profiling using STARmap PLUS analysis also revealed enrichment of DAA in TauPS2APP mouse cortex and hippocampus [101]. Interestingly, astrocyte proximity to Aβ plaques may vary between brain subregions; results from Stereo-seq analysis in human AD hippocampus revealed astrocyte enrichment nearby plaques in stratum lucidum, radiatum, and moleculare (SLRM) of the human hippocampus, whereas Aβ-associated astrocyte abundance was reduced in CA4 subregions [106]. In contrast to DAM and DAA, neurons overall were found to be depleted in proximity to Aβ plaques using STARmap PLUS in TauPS2APP AD mouse brain [101]. However, variations exist among neuron subtypes across regions. For example, an inhibitory neuron subtype, marked by *Sst* and *Chodl* genes, were found to have higher proportion nearby Aβ plaques in human cortical regions [151].

Beyond characterizing individual cell-type responses, spatial transcriptomics has enabled characterization of coordinated multicellular responses to Aβ plaques across cell types. For example, cells surrounding Aβ plaques featured DAM microglia within the inner core surrounding plaques, with DAA-like cells and oligodendrocyte precursors/oligodendrocytes localized to outer regions extending from Aβ plaques in TauPS2APP mouse brain [101]. DEG analysis in microglia, astrocytes, oligodendrocytes and neurons in proximity to plaques in TauPS2APP mouse brain revealed enrichment of genes involved in inflammation, gliogenesis, myelination and axon ensheathment [101].

Several studies reveal shared and distinct plaque-associated responses across varying genetic murine models of AD. In comparing humanized *APOE3* or *APOE4* knock-in mouse models crossed with 5xFAD animals, *APOE4*/5xFAD animals featured exacerbated upregulation of DAM/MGnD gene signatures by scRNA-seq analysis; subcluster analysis of Visium spots revealed *APOE4*/5xFAD enriched signatures featuring upregulation of reactive microglia signatures and genes related to microglia activation, synaptic pruning, neuronal apoptosis in *APOE4*/5xFAD animals compared to *APOE3*/5xFAD [102]. These studies highlight spatial sensitivity of microglia to pathological features such as Aβ plaques in AD pathology, which can be modulated by factors that enhance AD-onset and pathogenesis such as *APOE4*. Characterization of 5xFAD mice carrying the AD-associated *Trem2* R47H variant using MERFISH revealed region-specific cellular changes in proximity to Aβ, where DAM microglia in 5xFAD brain were higher in cortical layers V-VI compared to cortical layers II-III; 5xFAD/*Trem2* R47H also featured a slight decrease in DAM proportion compared to 5xFAD alone [76]. In human brain, triplication of APP on Chromosome 21 in DS individuals is thought to be causal to AD-like symptoms and early onset Aβ pathology [152]. Comparison of human AD and DS cortex by snRNA-seq and Visium spatial transcriptomic analysis revealed layer-specific changes in DEG profiles, and genes such as *CLU* and *VIM* were associated with regions enriched with Aβ plaques and fibrils [52]. Similarly, *Clu* and *Vim* were also found to be associated with amyloid in 5xFAD animals; other Aβ-associated genes such as *NEFH, NEFM, ALDOC* and *MAFB* are also found to show similar changes in 5xFAD and human AD/DS brain [52].

Transcriptional states in relation to tau pathology have also been defined using spatial transcriptomics; in contrast to Aβ and amyloid-related studies, fewer spatial transcriptomic studies have characterized profiles associated with tau. In TauPS2APP mouse hippocampus, ptau pathology is observed mainly in CA1 excitatory neurons and correlates with the local enrichment of oligodendrocytes [101]. In PS19 mouse brain, GeoMx DSP data analysis revealed region-specific transcriptomic changes in hippocampal CA3 subregion with up-regulation of genes involved in metabolic pathways before overt tau pathology [153]. DAM and DAA signatures emerge in parallel with the development of ptau pathology across multiple brain regions [153]. In human AD brain, analysis of the MTG region using the Visium platform identified gene signature profiles in regions enriched with AT8 ptau [100]. Visium spots containing AT8 ptau tangles and neuropil threads appeared to be enriched in upregulated genes/pathways associated with synaptic transmission, as well as downregulation of genes associated with apoptosis [100]. Further RNAscope analysis of human control and AD brain within the MTG region revealed elevation of *CSRP1* and *GLUL* in layer V astrocytes in proximity to neurofibrillary tangles (NFTs) and AT8 + ptau neuropil threads, and upregulation in microglia *C1QB* and *SPP1*, as well as *SNCG* neurons in layer II/III in proximity to both Aβ plaques and NFT/AT8 ptau pathology [100]. Recent characterization of NFT-bearing (AT8⁺) CA1 hippocampal neurons in late stage (Braak V–VI) AD using spatial transcriptomics [154] reported coordinated upregulation of synaptic genes (*VAMP2*, *SYT1*), molecular chaperones (*YWHAG*, *YWHAH*, *YWHAZ*), and genes associated with apoptosis, endoplasmic reticulum stress, and stress granule formation (*IFITM2*, *CCDC88B*, *PTMA*, *G3BP2*, and *ATF4*). These findings are consistent with prior observations from NFT-bearing (AT8⁺) neocortical neurons in BA9 late-stage AD (Braak VI) [18]. Notably, a similar transcriptional profile is observed in PART, another 3R/4R secondary tauopathy characterized by minimal Aβ deposits, suggesting that tau-driven transcriptional changes in hippocampal CA1 neurons may share transcriptional signatures with regions associated with low Aβ plaque deposition [154]. In contrast, spatial transcriptomics analysis of NFT‑rich (AT8⁺) areas in primary motor cortex from progressive supranuclear palsy (PSP, 4R primary tauopathy) brain, revealed a distinct transcriptional landscape marked by downregulation of genes involved in insulin signaling, phagocytosis, cellular stress, neutrophil degranulation, neurotransmitter release, oxidative phosphorylation, and NF‑κB signaling [155].

Emerging evidence suggests that cellular responses may be shaped not only by proximity to Aβ or tau individually, but in combination. Unique upregulated and downregulated genes and pathways have been characterized in human AD brain by spatial spots annotated for Aβ or tau pathology and Aβ/tau in combination using the Visium platform [100]. Regions with only tau pathology feature up-regulation of genes and pathways associated with synaptic transmission, and downregulation of genes and pathways associated with “negative regulation of apoptosis”. Interestingly, further co-expression analysis revealed that certain gene modules changed gradually as the distance from either Aβ or tau pathology increases, but similar gradual changes were not observed when considering both Aβ and tau in combination. These results suggest that combined analysis of Aβ and tau pathologies within the same tissue sections is important for distinguishing pathology-specific responses from combinatorial effects and to understand how overlapping pathological landscapes drive cellular dysfunction during AD progression.

### Spatially-resolved cell–cell interactions

In addition to cell-intrinsic responses to pathology, spatial transcriptomics has also allowed us to define how AD pathology reshapes communication or interaction profiles between neighboring cells. For example, knockdown of the dual-specificity phosphatase, *Inpp5d* (SHIP1) with nucleotide variants previously implicated in AD risk in PSAPP murine AD microglia increased plaque-associated microglia, and aggravated Aβ plaque burden [123]. Using the NICHES method [118] which can infer CCIs with spatial information, alterations were found in receptor-ligand pairs including genes involved in phagocytosis and lysosome activity (including C1q-Lrp1*,* C3-Lrp1, Timp1-Cd63, Pros1-Axl, and Ly86-Cd180) in cell clusters in close proximity to plaques and altered by *Inpp5d* depletion [123]. In human AD dorsal-lateral prefrontal cortex, downregulation of ligand-receptor pairs related to synaptic pathways was observed in regions enriched with glia [125]. Moreover, upregulation of ligand-receptor pairs related to immune response (CLU-TREM2, PLXNA1-TREM2, APOE4-LDLR and GFAP-APLNR) as well as the extracellular matrix and vesicle signaling was observed in regions enriched with Aβ [125]. Interestingly, transcriptomic changes indicative of synaptic degeneration were enriched in low Aβ regions, as well as dramatic changes in ligand-receptor pairs in low Aβ regions, suggesting that neurodegenerative effects are associated with areas with fewer Aβ plaques, and regions enriched with Aβ may be associated with late degeneration and cell death [125]. Targeted CCIs between reactive microglial and DAA-like astrocyte subclusters in Stereo-seq datasets from human AD hippocampus using StereoSiTE [119] revealed enrichment of ligand-receptor pairs involved in neuroinflammatory response such as SPP1-ITGB1 and MIF-CD44 in fimbria [106]. In APP^NL−G−F^ mouse brain, CCIs inferred using the CellPhoneDB method [117] on CosMx datasets revealed stronger receptor-ligand PIGs in proximity to plaques [105]. In particular, homotypic microglia-microglia DAM gene interactions such as Csf1-Csf1r and Cd44-Tyrobp (DAP12), as well as DAA astrocyte-microglia DAM interactions such as Apoe-Trem2, or Clu-Apoe were found to be enhanced in proximity to plaques [105]. In another study using APP^NL−G−F^ mouse brain, weighted gene co-expression network analysis [156] of multicellular spatial transcriptomic profiles also implicated that PIGs including secreted microglia DAM components such as *C1q* and *Apoe*, as well as DAM receptors such as *Trem2* and *Tyrobp* correlate with astrocyte activation profiles (*Gfap, C4, Clu, Prdx6, Cst3, Serpina3*) in the proximity of plaques [98].

Inferred CCI profiles can be stratified into regions or subregions. For example, comparison of cell–cell communication networks inferred by CellChat [112] in human DS and control brain revealed changes in CCIs in upper/lower cortical and white matter regions [52]. Interestingly, NECTIN signaling, a component required for synaptic maintenance [157–159] and implicated in genetic AD risk [160, 161] was found to be downregulated in DS and late-stage AD [52]. In transgenic R955-hTau mouse brain, BDNF neurotrophic signaling from DG in hippocampal regions was decreased compared to WT while neurotrophic signaling from cortical layer L2/3 to L4-6 was increased, suggesting a shift in neurotrophic signaling largely in the DG region in WT hippocampus to L2/3 cortex in R955-hTau animals [55].

Overall, use of spatial transcriptomics in AD research has uncovered important features in cell–cell communication networks that are impacted by pathology and vary across brain regions. However, CCI analysis is limited by the resolution and multiplexity of current spatial transcriptomic technologies. While imaging-based spatial transcriptomic technologies provide subcellular resolution for cell type-specific analysis, the limited gene panels constrain the number of CCIs that can be detected. Without full transcriptomic coverage, the interpretation of incomplete CCI networks can be potentially biased. Comprehensive characterization of CCI networks is in principle feasible using array-based spatial transcriptomic technologies with whole-transcriptomic data, but their multi-cellular resolution complicates interpretation due to limited cell-type specificity and reduced spatial precision (Table 2). A comprehensive and reliable characterization of CCI networks relies on the advancement of the spatial transcriptomics technologies towards simultaneous subcellular resolution and whole-transcriptomics coverage. Moreover, existing CCI inferences primarily rely on mRNA co-expression and physical proximity, lacking direct validation of the physical contact or functional interaction. Fundamentally, mRNA expression does not directly characterize post-transcriptional regulation, protein translation, secretion or receptor availability. In addition, spatial transcriptomics profiles frozen brain tissue, whereas CCIs are dynamic and temporally ordered. These limitations highlight the need to integrate spatial transcriptomics with complementary modalities, such as spatial proteomics and live imaging, to validate inferred CCIs.

### Spatial–temporal dynamics and disease progression

Although most spatial transcriptomic studies in AD rely on static tissue snapshots, emerging work suggests that molecular responses evolve dynamically across disease stages rather than remaining static. A major challenge in AD research is reconstructing how cellular states transition as pathology spreads across anatomically connected brain regions over time. Spatial transcriptomics is particularly well positioned to study this question because it preserves information on anatomical location, local pathology burden, and neighboring cellular states simultaneously.

Emerging evidence suggests that early disease stages are marked by well‑defined, cell‑type–specific transcriptional programs, whereas later stages are characterized by convergence of gene expression across cell types [162], reflecting widespread network disruption and roles for glial/neuronal communication [100, 163] with attenuated CCIs in AD normally involved in Aβ clearance [163]. In addition, accumulating evidence from spatial transcriptomic studies indicates that gene expression in Alzheimer’s disease is dynamically regulated, rather than uniformly up‑ or downregulated over disease progression. Instead, individual genes follow distinct, stage‑dependent trajectories characterized by transient phases of up‑ and downregulation. For example, the neuroprotective gene *PSAP* is upregulated in prefrontal cortex during early and intermediate stages of AD but progressively downregulated in advanced stages [163]. Such nonlinear expression patterns may help explain earlier, seemingly paradoxical observations of elevated synaptic gene expression—including *SNAP25*, *GABRA1*, *GRIN2B*, and *SYT1*—in ptau–bearing (AT8⁺) neurons undergoing tau‑mediated neurodegeneration [18], despite expectations of synaptic gene loss in degenerating neurons. These findings suggest that gene expression profiling performed at different disease stages may capture transient compensatory responses aimed at counteracting tau‑induced dysfunction. Consistent with this idea, other synaptic genes such as *SLC1A2*, *GRM7*, and *NRXN3* are upregulated at later stages of AD [163]. Why specific synaptic genes display distinct temporal sensitivities across disease stages remains a central open question in models of network vulnerability and pathological propagation. One plausible explanation is that expression of genes encoding synaptic receptors or ligands is dynamically shaped by changes in the availability of their cognate signaling partners, producing transcriptional responses that are inherently non–cell autonomous. Within vulnerable networks, tau-driven neurodegeneration may selectively disrupt afferent and efferent connections, altering synaptic input–output balance and depriving neurons of stabilizing circuit-level signals. Such connectivity loss could trigger compensatory transcriptional programs aimed at preserving synaptic function, excitability, or signaling capacity. In this framework, stage-dependent synaptic gene regulation reflects not only intrinsic neuronal stress responses but also evolving circuit context, which may in turn modulate susceptibility to further degeneration and constrain or facilitate the trans-synaptic propagation of pathology.

Given that spatial transcriptomics can characterize changes related to pathology in asymptomatic individuals, information from these studies may identify early prodromal events and cell‑specific signatures of vulnerability or resilience before they become obscured in advanced disease stages. Future efforts that integrate post‑mortem spatial transcriptomics with early‑stage human AD samples, longitudinal experimental models, and targeted perturbations will be critical for reconstructing disease trajectories and for defining molecular programs that drive, modify, or constrain neurodegeneration across AD stages.

### Insights and mechanisms in AD, lessons from spatial transcriptomics

With the rapid rise of studies utilizing spatial transcriptomics in AD, an immense amount of data linking region-specific, pathology-associated and pathology-independent features is being accrued. Although these results can only offer a snapshot of region and/or pathology-associated dysfunction in human and mouse brain (Table 3), some underlying trends in AD have been established through spatial transcriptomics thus far.

First, glial responses to pathology are highly spatially restricted rather than uniformly distributed across affected tissue. Microglia and astrocytes consistently adopt pathology-associated states in close proximity to Aβ plaques, suggesting that local pathological microenvironments are major drivers of reactive state transition. Second, neuronal vulnerability is highly selective across brain regions and cell subtypes, which can be further linked to neuronal degeneration during AD progression. Spatial transcriptomics studies indicate that CA1 neurons are particularly vulnerable while CA3/CA4 neurons show attenuated degeneration [106], which are consistent with previous findings that hippocampal CA1, entorhinal cortex and subiculum subregions are particularly vulnerable during AD pathogenesis [164], and during early stages of degeneration, CA2, CA3 and hilus regions are relatively resistant to degeneration [165]. This implicates progressive heterogeneity in neuronal dysfunction during AD onset. Third, consistent disruption of local intercellular communication networks involved in synaptic maintenance, immune signaling, and Aβ clearance is observed in AD brain. Finally, genetic modifiers such as *APOE*, *TREM2*, and *INPP5D* further shape pathology-associated cellular responses, highlighting how inherited risk factors may influence local disease microenvironments. Use of mouse models has also demonstrated alterations in expression through modulation of genes related to AD risk such as *APOE4* in 5xFAD mouse models [102]; some models such as *Inpp5d* depletion in PSAPP animals show effects in enhancing microglia recruitment to plaques [123], while others such as *Trem2* R47H in 5xFAD brain show only modest effect in plaque targeting [76].

Although definitive roles for specific expression of genes and their contribution to degenerative phenotypes will require further mechanistic evidence, spatial transcriptomics so far has demonstrated potential versatility in defining degenerative events in certain regions and cellular subtypes.

## Limitations, challenges and future directions with spatial transcriptomics

Here, we discuss both experimental challenges in implementing spatial transcriptomics in AD brain tissues and computational challenges with spatial transcriptomic data analysis in AD.

### Challenges related to AD brain tissue and tissue processing

Use of postmortem tissue in spatial transcriptomics can be challenging, especially where variables such as postmortem interval (PMI) can affect RNA integrity and downstream detection in both human [166, 167] and mouse brain [168]. RNA Integrity Number (RIN) is a commonly used metric to assess RNA quality[169, 170], with many spatial transcriptomic platforms requiring a RIN value greater than 7 for optimal performance. Studies have shown a negative correlation between RIN and PMI and have observed selective degradation of certain mRNA species, particularly neuronal synaptic mRNA as RIN declines [169]. Long-term storage of brain tissue can also impede RNA detection by affecting probe binding to target molecules [171]. Autofluorescence signals in aged brain tissue can also interfere with image acquisition; lysosomal accumulation of lipofuscin lipid, protein, sugar and ion aggregates with age is particularly problematic [172]. Photobleaching using light-emitting diode (LED)-based [173] or commercial Sudan Black-based reagents (TrueBlack Plus) [174] can be used to reduce lipofuscin-derived autofluorescence.

### Spatial transcriptomics and challenges related to technology and analysis in AD

#### Considerations with current spatial transcriptomic platforms

Despite increasing use of spatial transcriptomics in AD, current spatial transcriptomic platforms face substantial limitations that warrant further improvement. A major limitation associated with commercialized platforms used in AD research is the trade-off between multiplexity and resolution. Array-based spatial transcriptomic technologies such as Visium offer whole-transcriptomic profiling without subcellular resolution and therefore lack cell type-specific analysis. *In situ* spatial transcriptomic methods such as MERFISH, Xenium and CosMx can achieve subcellular resolution but with limited multiplexity. Emerging efforts to develop methods to profile whole-transcriptome at subcellular resolution suffer from technical issues including low detection rates with increased resolution for array-based methods, and increased error rates with increased multiplexity for imaging-based methods. For example, Stereo-seq can detect transcripts at subcellular resolution (~ 0.5 μm) and can theoretically profile whole transcriptomic gene expression. However, Stereo-seq is limited to detection of ~ 300–400 genes per cell/nucleus in human brain samples [106, 163], and fewer than 700 genes detected per cell in mouse brain [175]. To address this, spots detected by Stereo-seq can be aggregated into larger bins (e.g., 50 μm x 50 μm); while this can increase overall detection to 834 genes per bin in human hippocampus, subcellular resolution is lost by aggregating spot profiles [106]. Given the spatial resolution and transcript coverage of array-based and imaging-based methods, array-based methods are suited for discovery-based applications, while imaging-based methods may be more compatible with validation studies (Table 2).

Thus, there is a need to simultaneously improve both multiplexity and resolution to enable true whole-transcriptome and subcellular-resolution profiling. Additionally, FFPE compatibility for postmortem human brain samples and pathology staining compatibility are also critical in the analysis of AD brain tissue (Table 1). Current spatial transcriptomic experiments are expensive, and limited in throughput, scalability and tissue coverage. Substantial effort is needed to develop cost-effective spatial transcriptomic platforms that can generate high quality data for large cohorts.

#### Limitations related to computational analysis

Current computational pipelines used in spatial transcriptomics are presented with challenges with cell segmentation, cell type deconvolution and annotation, and in modeling spatial microenvironments. Both subcellular-resolution array-based and imaging-based spatial transcriptomic platforms rely on accurate cell segmentation to generate cell-level gene expression profiles. Cell segmentation in AD brain tissues is particularly difficult due to high cell density in the brain, irregular cell morphology and high background levels from neuropil and AD pathology. Ambiguous and unexpected gene expression profiles have been associated with inaccurate cell segmentation [76]. To improve cell segmentation accuracy, emerging efforts integrate morphological images, transcript distribution and gene expression profiles from reference scRNA-seq datasets to optimize cell segmentation pipelines [72, 176, 177].

Cell deconvolution and annotation are essential for cell type-specific analysis. For multicellular array-based spatial transcriptomic datasets, cell deconvolution is needed to infer cell type composition in each multicellular spot. Accurate deconvolution of cell populations requires high-quality reference scRNA-seq datasets with strong congruency with respect to cell types and signatures with spatial transcriptomic profiles. For subcellular spatial transcriptomic data, accurate annotation of cell populations is challenging due to the limited number of genes profiled. For both cell type deconvolution and annotation, scRNA-seq profiles in matched samples can improve accuracy by reducing errors due to biological heterogeneity across samples [178, 179]. Additionally, batch correction may be needed to combine and integrate profiles from scRNA-seq and spatial transcriptomic platforms [180, 181].

Modeling spatial microenvironments in AD brain involves the characterization of cellular response in the vicinity of AD pathology. Computational challenges arise in identification and segmentation of AD pathology, and associating pathology with gene expression. Various methods have been used to annotate pathology, including use of existing image processing software with custom modules, traditional image segmentation algorithms and implementing machine learning-based segmentation methods. Due to substantial variations in sample quality, pathological morphology, staining strategies, and imaging platforms, the field currently lacks a robust computational pipeline that can be used across a variety of datasets. Standardized procedures to establish relationships between pathological microenvironments and transcriptomic features require future development of toolkits and pipelines that streamline workflow from pathology segmentation to downstream analysis in spatial microenvironments.

### Future directions

The full impact of spatial transcriptomics in Alzheimer’s disease is likely to emerge through its integration with other complementary data modalities. Spatial transcriptomics has provided a critical framework for linking gene expression with histological context, anatomical organization, and pathological hallmarks across brain regions. However, AD pathobiology is governed by regulatory mechanisms that extend beyond transcriptional states alone. Integration with spatial epigenomic [182–189] and proteomic data [190, 191] offers an opportunity to capture regulatory architecture and protein-level signaling within pathological microenvironments in the AD brain. Spatial multi-omic integration may enable a more mechanistic interpretation of how pathological niches influence cellular states and intercellular interactions during AD progression. As cohort sizes continue to increase, integration of spatial transcriptomic profiles with genetic data is becoming increasingly feasible. Although genome-wide association studies have identified numerous AD risk loci, spatial transcriptomics can contextualize these variants within specific cell types, brain regions, and pathological microenvironments. Realizing this potential will require the development of computational frameworks capable of jointly modeling heterogeneous spatial data types [192, 193], genetic profiles, pathology features, and regulatory hierarchies within complex brain tissue.

Although efforts have been made to standardize spatial transcriptomic data analysis [91, 194], there is a need to standardize pathology‑associated computational pipelines for spatial transcriptomic analysis tailored for AD studies. Variability in tissue preservation, staining strategies, spatial transcriptomic platform-specific biases, cell segmentation accuracy, and pathology morphology currently limits cross‑study comparability. Community‑driven benchmarks, curated reference datasets, and shared annotation frameworks could improve reproducibility and facilitate quantitative comparison of pathology‑associated cellular states across cohorts and platforms. Looking forward, pipeline building and execution can be accelerated with use of artificial intelligence (AI) agent platforms or tools [195, 196] powered with large language models (LLM). Recent advances in agentic bioinformatics systems can autonomously orchestrate multi-step analytical workflows, interface with specialized bioinformatics tools, and adaptively generate reproducible pipelines. The combination of curated standard pipelines by bioinformatics experts and adaptive applications by AI agentic systems can also enable rapid iteration and benchmarking of pathology-aware spatial transcriptomic pipelines, particularly as community standards and reference datasets continue to mature. Though AI agent platforms allow natural language specifications which greatly improve the ease of use, they inherit the primary limitations of LLM particularly hallucinations, therefore supervision and intervention from bioinformatics experts are necessary for current AI agent tools to avoid inappropriate analysis workflows resulting in misleading biological findings [197, 198].

Although spatial transcriptomics captures static snapshots of brain tissue, AD manifests through spatially and temporally coordinated pathological progression across brain regions. Future efforts should therefore focus on modeling spatial–temporal disease trajectories by integrating spatial transcriptomic and pathological profiles to build computational frameworks that infer temporal ordering across regions, pathological burdens and disease stages. Existing efforts have demonstrated promising progress on modeling spatial and temporal gene expression patterns using spatial transcriptomics data. For example, trajectory inference methods such as SpaTrack [199] leverages optimal transport to integrate spatial and transcriptomic information across multiple samples over temporal intervals, enabling reconstruction of dynamic changes in cell state and tissue architecture over time. Specifically, modeling AD progression requires coupling of the pathological profiles into spatial–temporal models. Using these models, development of AD pathology can be inferred by cellular level changes including cell vulnerability, resilience and gene expression patterns across regions. Essentially, spatial–temporal modeling can reconstruct dynamic cellular state transitions and evolving pathological niches along the course of AD progression. Further integrating with longitudinal imaging and cerebrospinal fluid (CSF) data may provide insights into biomarkers for non-invasive clinical diagnosis.

Ultimately, the potential translational contribution of spatial transcriptomics in AD lies in its ability to anchor molecular alterations to defined anatomical regions and pathological contexts, providing a biological bridge between cellular mechanisms and clinically observable phenotypes. By resolving region- and pathology-specific cellular programs, spatial transcriptomics studies can inform the interpretation of in vivo imaging signals such as positron emission tomography (PET) imaging and magnetic resonance imaging (MRI), as well as CSF or plasma biomarkers, by linking them to their underlying cellular and molecular origins [106, 127]. As spatial datasets expand across cohorts and disease stages, these context-resolved molecular profiles may support improved patient stratification, guide target prioritization towards pathology-relevant cellular niches, and inform the design of stage- or region-specific therapeutic strategies. While spatial transcriptomics is not itself a clinical tool, its translational value lies in providing a mechanistic framework that connects molecular pathology with biomarkers and disease progression in AD.

Collectively, these future directions highlight how spatial transcriptomics in AD moves beyond descriptive atlases toward an integrative framework for modeling disease-relevant molecular process in their native tissue context. Advances in spatial resolution, multiplexity, and pathology awareness, together with improved standardization, computational modeling and multi-omic integration, are expected to expand the analytical scope of spatial transcriptomic studies beyond static atlases. By enabling pathology-aware cellular profiling, spatial–temporal inference, and translational anchoring to imaging readouts and fluid biomarkers, spatial transcriptomics is playing an increasingly important role in connecting molecular pathology with disease progression and clinical phenotypes.

## Conclusions

Over the past century, AD research has progressed from largely descriptive characterization of neuropathological features to increasingly detailed investigation of the molecular and cellular processes that underlie disease progression. Spatial transcriptomics represents an important step in this trajectory by enabling gene expression to be examined directly within intact tissue preserving anatomical organization and pathological context. As highlighted in this review, spatial transcriptomic studies have provided insight into region-specific vulnerability and resilience, cellular responses to Aβ and tau pathology, and spatially resolved cell–cell interactions and spatial–temporal trajectories that are difficult to capture using bulk RNA sequencing or dissociated single-cell approaches alone.

Despite these advances, current spatial transcriptomic technologies are limited by fundamental trade-offs between spatial resolution and multiplexity. Imaging-based platforms can achieve subcellular spatial resolution but are typically limited to a few hundred up to several thousand genes. In contrast, array-based approaches offer whole transcriptome coverage but often measure signals derived from multiple cells within each capture location. As a result, integration with scRNA-seq data has become a common strategy to accommodate limited spatial resolution or multiplexity, facilitate cell type annotation, and enable in-depth investigation of cell type-specific spatial patterns in AD brain. At the same time, computational challenges remain substantial, including accurate cell segmentation, reliable identification of pathological structures, and modeling gene expression in densely packed and structurally complex regions of the diseased brain.

Further progress in the field will likely depend on continued improvements in both experimental and analytical approaches. Technical advances that increase spatial resolution while maintaining whole transcriptome coverage, improve compatibility with FFPE tissue and histopathological staining will expand the scope of spatial transcriptomic studies in AD. In parallel, there is a need for standardized analytical frameworks that incorporate pathological features and support reproducible comparisons across platforms, cohorts, and disease stages. Integration of spatial transcriptomics with complementary modalities, including histopathological imaging data, spatial proteomics, spatial epigenomics, and genetic variation, may help connect molecular alterations to regulatory mechanisms and disease trajectories within defined brain microenvironments.

The potential translational value of spatial transcriptomics arises from its ability to anchor molecular and cellular changes to specific anatomical regions and pathological context. By providing a framework that connects regional cellular programs with clinically observable phenotypes, spatial transcriptomic studies may improve interpretation of neuroimaging and fluid biomarkers, support more refined patient stratification, and aid identification of cellular processes that represent potential therapeutic targets. As the field moves beyond descriptive mapping toward integrative and longitudinal analyses of disease progression, spatial transcriptomics is likely to play an increasingly important role in clarifying the cellular mechanism of AD and informing future therapeutic strategies.

Over a century has passed since Alois Alzheimer first described [200–203] unique symptoms and brain pathology from a patient, Auguste Deter, who had suffered from irregular memory deficits at the 37th meeting of South-West German psychiatrists in Tübingen, Germany in 1906 [203]. It is likely that Alzheimer had used a simple Zeiss light microscope mounted on a jug-handle stand, considered to be state-of-the-art at the time, which allowed him to publish a more complete work with illustrations of AD pathology in 1911 [201]. Although presentation of his work in 1906 solicited no questions or discussion and was initially deemed to be “unsuitable for publication” in the meeting proceedings [203], the meeting organizers later published a two-page summary describing Alzheimer’s clinical and pathological findings in 1907 [200, 202] which would shape and define the course of AD research to this day.

As spatial transcriptomic technology continues to evolve, the AD field will surely reap the benefits of this growing technology. Although the cost of spatial transcriptomics remains high, perhaps we can be encouraged by Alzheimer who for years, worked without salary in Munich and at times, maintained his research from his personal funds [203]. Passion and dedication in the AD field is commonplace, and with Alois Alzheimer as a leading example, pathologists, engineers and computational biologists will surely find a way forward for AD and related dementias.

## Data Availability

No datasets were generated or analysed during the current study.

## Abbreviations

Aβ: Amyloid beta
AD: Alzheimer’s disease
AI: Artificial intelligence
CA: Cornu Ammonis
CCI: Cell-cell interaction
CCF: Common coordinate framework
CSF: Cerebrospinal fluid
DAA: Disease-associated astrocyte
DAM: Disease-associated microglia
DEG: Differentially expressed gene
DG: Dentate gyrus
DNB: DNA nanoballs
DS: Down syndrome
FFPE: Formalin-fixed paraffin-embedded
H&E: Hematoxylin and eosin
IF: Immunofluorescence
ISH: In situ hybridization
ISS: In situ sequencing
MCI: Mild cognitive impairment
MERFISH: Multiplexed Error-Robust Fluorescence In Situ Hybridization
MGnD: Neurodegenerative microglia
MRI: Magnetic resonance imaging
MTG: Middle temporal gyrus
NGS: Next-generation sequencing
NFT: Neurofibrillary tangle
PART: Primary age-related tauopathy
PET: Positron emission tomography
PIG: Plaque-induced gene
PMI: Postmortem interval
PSP: Progressive supranuclear palsy
ptau: Phosphorylated tau
ROI: Region of interest
scRNA-seq/snRNA-seq: Single-cell RNA sequencing/single-nucleus RNA sequencing
SLRM: Stratum radiatum, lacunosum, and moleculare
STARmap: Spatially Resolved Transcript Amplicon Readout Mapping
Stereo-seq: SpaTial Enhanced Resolution Omics-sequencing
WM: White matter
